# Genistein and Resveratrol: Inhibitors of Kv1.3 Channels in Cancer Cells

**DOI:** 10.3390/membranes16050159

**Published:** 2026-04-30

**Authors:** Andrzej Teisseyre, Anna Uryga, Kamila Środa-Pomianek, Anna Palko-Labuz

**Affiliations:** Department of Biophysics and Neurobiology, Wrocław Medical University, ul. Chałubińskiego 3a, 50-368 Wrocław, Polandkamila.sroda-pomianek@umw.edu.pl (K.Ś.-P.);

**Keywords:** Kv1.3 channel, Jurkat T cell, genistein, resveratrol, cancer cell proliferation, cancer cell apoptosis

## Abstract

Background: Genistein and resveratrol are bioactive compounds isolated from plants, recognized for their diverse biological activities including anti-cancer properties. Both compounds are also known as modulators of various types of ion channels, including voltage-gated potassium channels, Kv1.3. These channels are widely expressed in normal and cancer tissues. Their activity is crucial in regulating cell proliferation and apoptosis in cells that express Kv1.3 channels. The potential clinical application of channel inhibitors may extend to treating cancers characterized by an overexpression of these channels. Methods: This study investigates the inhibitory effects of genistein and resveratrol on Kv1.3 channels expressed in the cancer cell line Jurkat T by applying a whole-cell patch clamp. Results: Applying both compounds at concentrations ranging from 3 μM to 90 μM leads to a dose-dependent inhibition of channel activity, reducing it to approximately 50% of the control level. This inhibitory effect was reversible and associated with a significant reduction in the activation rate. When combined with simvastatin, the inhibitory effect exhibited synergy; however, it was additive when co-applied with mevastatin. Conclusions: The channel inhibition may putatively be linked to the anti-cancer activities of these compounds on Kv1.3 channel-expressing cancer cells, especially when co-applied with the statins.

## 1. Introduction

**Figure 1:**
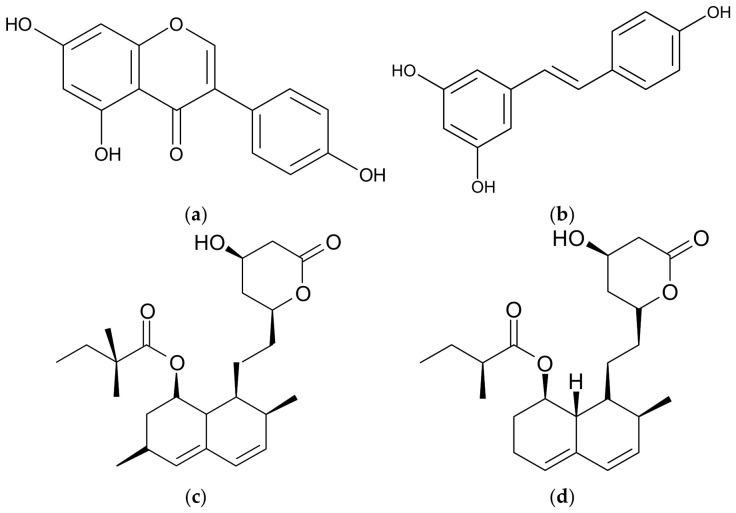
Structural formulas of genistein (**a**), resveratrol (**b**), simvastatin (**c**) and mevastatin (**d**).

Genistein (Figure 1a) is a plant-derived isoflavone found in several vegetables, recognized primarily as an effective inhibitor of protein tyrosine kinase (PTK). It has also been identified as a naturally occurring anti-cancer agent [1]. Additionally, genistein inhibits various ion channels, including several types of potassium channels [2]. Importantly, its inhibitory action on these channels occurs via a PTK-independent mechanism likely involving direct interactions with channel proteins [2].

Resveratrol (3,4′,5—trihydroxystilbene—Figure 1b) is a biologically active polyphenol isolated from plants—most notably present in red grapes and red wine—exerting various biological effects, including anti-cancer activity [3]. Like genistein, resveratrol modulates different potassium channel activities [2].

The voltage-gated potassium channel Kv1.3, encoded by the KCNA3 gene, is distributed across diverse tissues [4] and can be found not only within plasma membranes but also embedded within inner mitochondrial membranes (mito Kv1.3) [5]. The functionality of these channels plays an essential role in regulating cellular proliferation and apoptosis among cells that express them [4,5,6,7,8,9,10,11,12,13,14]. Significant alterations in their expression may occur during certain cancer conditions [6,7,8,9,10,11,12,13,14].

Inhibitors targeting these channels hold promise for clinical applications aimed at treating various disorders—including specific cancers like melanoma, pancreatic ductal adenocarcinoma (PDAC), multiple myeloma and B-type chronic lymphocytic leukemia (B-CLL)—that exhibit an overexpression of Kv1.3 channels [6,7,8,9,10,11,12,13,14,15,16,17,18,19,20,21].

Research suggests that some lipophilic organic inhibitors of Kv1.3 channels may possess anti-proliferative and pro-apoptotic properties against cancers expressing these channels while sparing normal cells [6,7,8,9,10,11,12,15,16,17,18,19,20].

Among these inhibitors are certain flavonoids, chalcones and statins [12,17,19], the efficacy of which can be enhanced when combined with statins, such as simvastatin or mevastatin [17,19]. The augmented channel inhibition may be correlated to improved pro-apoptotic activity on Kv1.3 channel-expressing cancer cells of Jurkat T [17,19].

Previous studies have established that both genistein and resveratrol inhibit Kv1.3 channels in human T cells via concentration-dependent mechanisms accompanied by significant reductions in activation rates without altering the kinetics of inactivation [12].

It is well-known that the function of membrane proteins, including ion channels, may change in cancer cells when compared to normal cells. Our previous studies have shown that sensitivity of Kv1.3 channels expressed in cancer cells of Jurkat T to inhibition by naturally occurring compounds, including resveratrol, may be lower than in the case of channels in normal human T cells [Gąsiorowska—unpublished observations]. However, the inhibitory effect of resveratrol on Kv1.3 channels in cancer cells was not studied in detail. The influence of genistein on the activity of Kv1.3 channels in cancer cells still remains unknown.

The objective of this study was to investigate the influence of genistein and resveratrol on the activity of Kv1.3 channels in cancer cells. Both compounds were applied alone and in combination with simvastatin and mevastatin (Figure 1c,d). The study was performed on a relevant model system—Kv1.3 channels expressed in the human leukemic cell line Jurkat T [22,23].

Our findings demonstrate that both genistein and resveratrol inhibit Kv1.3 channels in Jurkat T cells; however, their effects are less pronounced than those observed previously in normal human T lymphocytes [12]. Notably, the combined application of each compound with the statins resulted in significantly augmented inhibitory effects compared to an individual application of each compound alone.

## 2. Materials and Methods

### 2.1. Cell Culture and Solutions

Jurkat (clone E6-1) human leukemic T cells were procured from the American Type Culture Collection (Manassas, VA, USA) and cultured in RPMI 1640 medium (Sigma-Aldrich, St. Louis, MO, USA) supplemented with 10% heat-inactivated FBS, 10 mM HEPES and glutamate (2 mM). Cultures were maintained at 37 °C under a humidified atmosphere containing 5% CO_2_. During the experiments, cells were placed in the external solution, which contained: 150 mM NaCl, 4.5 mM KCl, 1 mM CaCl_2_, 1 mM MgCl_2_, 10 mM HEPES, 10 mM Glucose, and pH = 7.35, adjusted with NaOH. The internal (pipette) solution contained: 150 mM KCl, 1 mM CaCl_2_, 2 mM MgCl_2_, 10 mM HEPES, 10 mM EGTA, and pH = 7.2, adjusted with KOH. The calculated concentration of free calcium ions in the internal solution was below 100 nM [24]. This concentration was below the activation threshold of calcium-activated potassium channels K_Ca_2.2, which are also expressed in Jurkat T cells [24,25]. Activation of these channels would produce an additional potassium current, irrelevant for this study [25]. The chemicals were purchased from the Polish Chemical Company (POCH, Gliwice, Poland), except for HEPES and EGTA, which were purchased from SIGMA. Genistein and resveratrol were purchased from Alexis Biochemicals (Lausen, Switzerland).

### 2.2. Patch-Clamp Recordings

Dishes with cells were placed under an inverted Olympus IMT-2 microscope. Solutions containing tested compounds were applied using the eight-channel gravitation perfusion system (ALA Scientific Instruments, Farmingdale, NY, USA). Pipettes were pulled from borosilicate glass (Hilgenberg, Malsfeld, Germany). The pipette resistance was higher than 3 MΩ.

Whole-cell potassium currents in TL were recorded by applying the patch-clamp technique [26]. The currents were recorded using an EPC-7 Amplifier (HEKA, Lambrecht, Germany), low-pass-filtered at 3 kHz, and digitized using a CED Micro 1401 analog-to-digital converter (Cambridge, UK) with a sampling rate of 10 kHz. The influence of selected compounds on the activity of the channels was studied by applying the voltage ramp protocol. Voltage ramps gradually depolarized cell membranes from −100 mV up to +40 mV. The time duration of a single “ramp” was 340 ms, and the time interval between successive “ramps” was 30 s. For the time between “ramps”, examined cells were kept at a holding potential of −90 mV. Upon application of the voltage ramp protocol, potassium currents in Jurkat T cells could be stably recorded for at least 20 minutes after “break-in” to the whole-cell configuration. During the offline analysis, the value of Kv1.3 currents at the end of a voltage ramp (+40 mV) was calculated. For this purpose, the leak current estimated at +40 mV was subtracted from the total ramp current recorded at this voltage.

In order to study the influence of selected compounds on the channel activation and inactivation kinetics in more detail, another protocol of depolarizing voltage stimuli was applied. This protocol contained depolarizing voltage steps from the holding potential of −90 mV to +60 mV (500 ms step duration) applied every 30 s.

All experiments were carried out at room temperature (22–24 °C).

Unless otherwise stated, the data are presented as mean ± standard deviation.

### 2.3. Data Analysis

The inhibition of the channel is presented in terms of a relative current recorded upon application of the studied compounds, defined as I/I_contr_; here, I—Kv1.3 currents upon application of an examined compound at +40 mV, and I_contr_—Kv1.3 current recorded on the same cell at +40 mV under control conditions. Activation kinetics were measured in terms of the time-to-peak (T_peak_). The higher the value of T_peak_, the slower the activation kinetics were. Since the T_peak_ values varied remarkably among examined cells, slowing of the activation kinetics upon application of the compounds was measured in terms of a relative kinetics slowing (RKS), defined as follows:

RKS = ((T_peakcompound_ − T_peakcontrol_)/T_peakcontrol_) × 100% (1)

where

T_peakcompound_—mean time-to-peak value upon an application of the compound [ms].

T_peakcontrol_—mean time-to-peak value under control conditions [ms].

Inactivation kinetics were fitted by a single exponential function, defined as follows:

I(t) = A_1_ × exp(−t/τ) + A_2_ (2)

where A_1_ and A_2_—fit constants; t—time; and τ—inactivation time constant [ms].

The τ value is the measure of the inactivation rate. The slower the inactivation, the higher the value of τ.

Statistical analysis was performed by applying Student’s unpaired *t*-test or one-way ANOVA test. The results were considered statistically significant when *p* < 0.05.

## 3. Results

**Figure 2:**
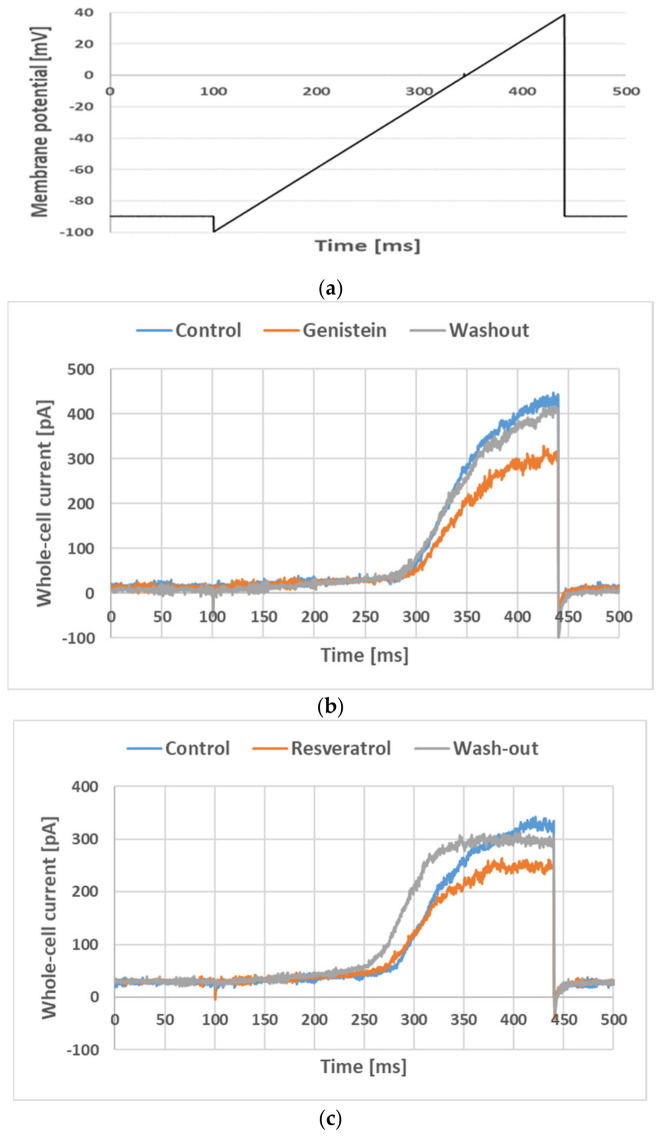
Whole-cell currents recorded in Jurkat T cells (**b**,**c**) applying the “voltage ramp” (**a**) under control conditions, upon an application of 30 µM genistein (**b**), 30 µM resveratrol (**c**) and during wash-out of the drugs.

Figure 2 depicts examples of the whole-cell ramp currents recorded in a Jurkat T cell with the voltage ramp applied (as defined in Section 2, Figure 2a) under control conditions, upon application of genistein and resveratrol at a concentration of 30 µM and after wash-out of the compound (Figure 2b and Figure 2c, respectively).

The figure depicts raw ramp currents (without leak subtraction), which contained two components: small linear and much bigger non-linear ones. The linear component was an unspecific leak current. This current was irrelevant for this study, and it was subtracted from the total current during the offline analysis. On the other hand, the non-linear component was due to an activation of Kv1.3 channels [27]. Visibly, an application of both genistein and resveratrol significantly diminished the amplitude of the Kv1.3 currents. The current recovered after the wash-out of genistein and resveratrol. This indicates that both compounds exerted an inhibitory effect on the current and this effect was fully reversible.

**Figure 3:**
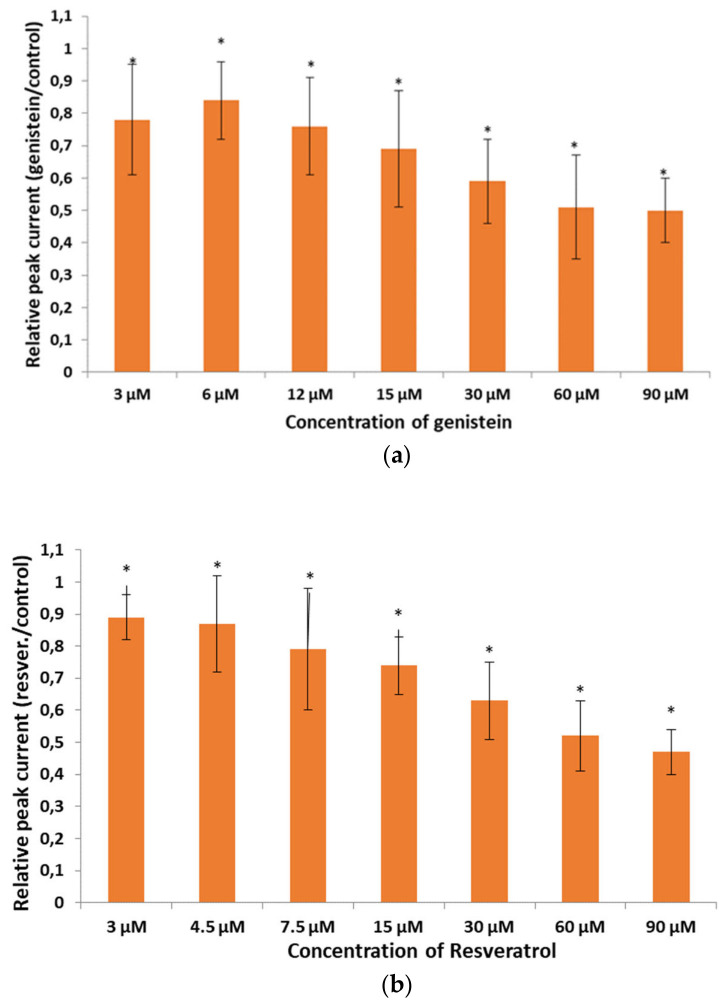
Relative peak current upon application of genistein (**a**) and resveratrol (**b**) at different concentrations. Error bars are shown. *—statistical significance.

The inhibitory effects of genistein and resveratrol on Kv1.3 currents in Jurkat T cells were studied at different drug concentrations. Relative peak currents upon application of both compounds at various concentrations from 3 µM to 90 µM are shown in Figure 3. Upon application of genistein at concentrations of 3 µM, 6 µM, 12 µM, 15 µM, 30 µM, 60 µM and 90 µM, the currents were reduced to: 0.78 ± 0.17 (n = 17), 0.84 ± 0.12 (n = 18), 0.76 ± 0.15 (n = 21), 0.69 ± 0.18 (n = 18), 0.59 ± 0.13 (n = 13), 0.51 ± 0.16 (n = 21) and 0.50 ± 0.10 (n = 20) of the control value, respectively (Figure 3a, Supplementary Table S1). The inhibitory effect of genistein on the currents was statistically significant (*p* < 0.05) at all the concentrations applied and it was also weakly dose-dependent (Figure 3a). An application of resveratrol at concentrations of 3 µM, 4.5 µM, 7.5 µM, 15 µM, 30 µM, 60 µM and 90 µM reduced the currents to: 0.89 ± 0.07 (n = 19), 0.87 ± 0.15 (n = 19), 0.79 ± 0.19 (n = 21), 0.74 ± 0.09 (n = 24), 0.63 ± 0.12 (n = 11), 0.52 ± 0.11 (n = 11) and 0.47 ± 0.07 (n = 20) of the control value, respectively (Figure 3b, Supplementary Table S2). The inhibitory effect of resveratrol on Kv1.3 currents in Jurkat T cells was statistically significant (*p* < 0.05) at all the concentrations and weakly dose-dependent (Figure 3b).

**Figure 4:**
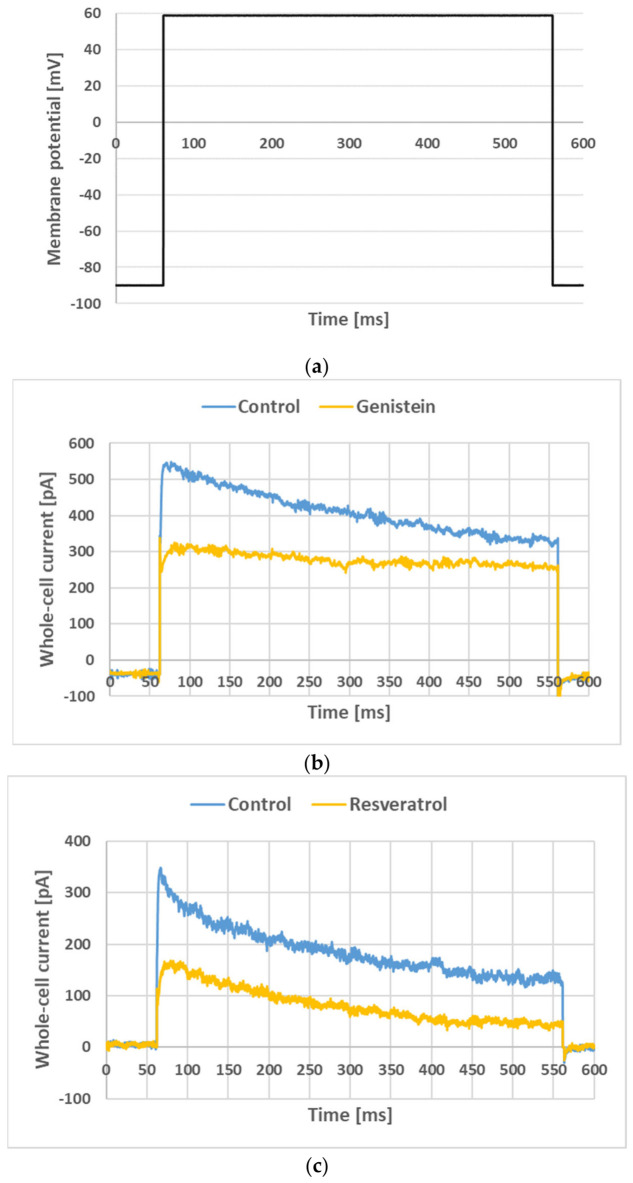
Whole-cell currents recorded in Jurkat T cells (**b**,**c**) applying the voltage step (**a**) under control conditions and upon an application of 30 µM genistein (**b**) or 90 µM resveratrol (**c**). Records upon wash-out of the compounds were omitted for clarity.

Our previous studies performed on Kv1.3 channels in normal human T lymphocytes demonstrated that the application of genistein and resveratrol significantly slowed down the channel activation rate without significantly changing the inactivation rate [12]. Therefore, it has been studied whether slowing down also occurs in the case of the channels expressed in cancer cells. Figure 4 shows the currents recorded when applying the voltage step protocol (see Section 2, Figure 4a) under control conditions and upon application of genistein at a concentration of 30 µM (Figure 4b) and resveratrol at a concentration of 90 µM (Figure 4c).

**Figure 5:**
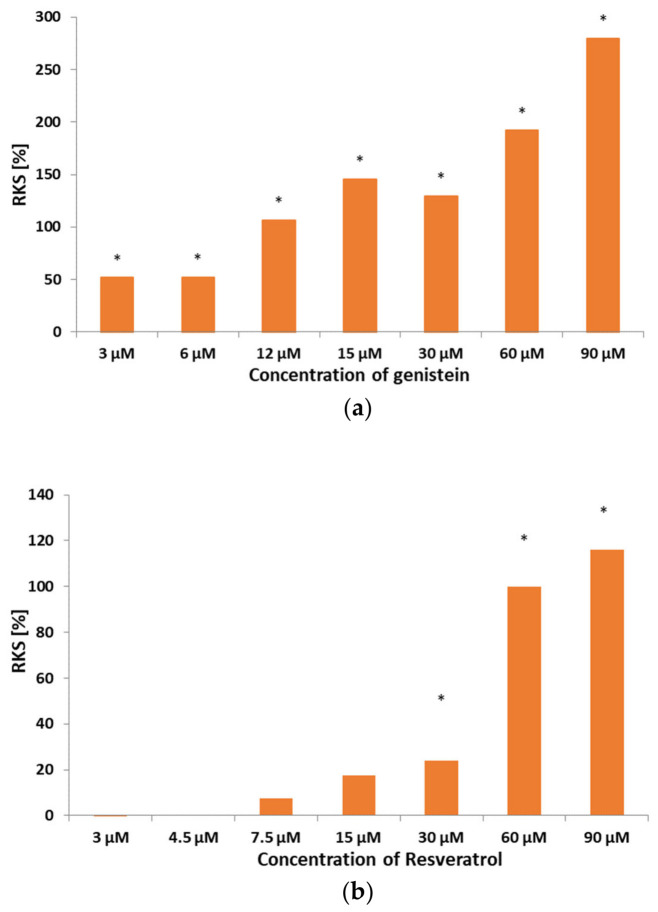
Relative kinetics slowing (RKS—defined in Section 2) upon an application of genistein (**a**) and resveratrol (**b**) at different concentrations. *—statistical significance.

Remarkably, the application of both compounds significantly slowed down the activation rate of the currents in Jurkat T cells (Supplementary Tables S3 and S4). The slowing down of the activation rate was measured in terms of the relative kinetics of slowing (RKS), defined by Formula (1) in Section 2. Figure 5 depicts the RKS values calculated for the currents recorded upon the application of genistein and resveratrol at different concentrations (Figure 5a,b). In the case of genistein application, slowing down of activation kinetics was statistically significant (*p* < 0.05) at all applied concentrations (Figure 5a). Activation kinetics slowing down upon application of resveratrol were statistically significant (*p* < 0.05) only at concentrations of 30 µM, 60 µM and 90 µM (Figure 5b). Apparently, RKS values were higher when genistein was applied rather than when resveratrol was applied.

It was also studied whether an application of genistein and resveratrol changed the inactivation rate, measured in terms of the inactivation time constant τ calculated by applying Equation (2) (see Section 2 and Supplementary Table S5). Upon application of genistein at a concentration of 30 µM, the τ value was equal to 205.8 ± 40.62 ms (n = 16). This value was not significantly different (*p* > 0.05) from the control value of 201.6 ± 28.07 ms (n = 16). Lack of change in the inactivation rate was also observed at other concentrations of this compound. Upon application of resveratrol at a concentration of 90 µM, the τ value was equal to 175.4 ± 25.97 ms (n = 16). This value was not significantly different (*p* > 0.05) from the control value of 178.89 ± 30.69 ms (n = 14). The lack of influence of resveratrol application on the inactivation kinetics was also observed at lower concentrations of this compound.

Our previous study has shown that the inhibitory effects of genistein and resveratrol on Kv1.3 channels in normal human T lymphocytes are additive [12]. Therefore, it is of interest to prove whether additive inhibition of the channels also occurs in the case of the co-application of genistein and resveratrol in cancer Jurkat T cells.

**Figure 6:**
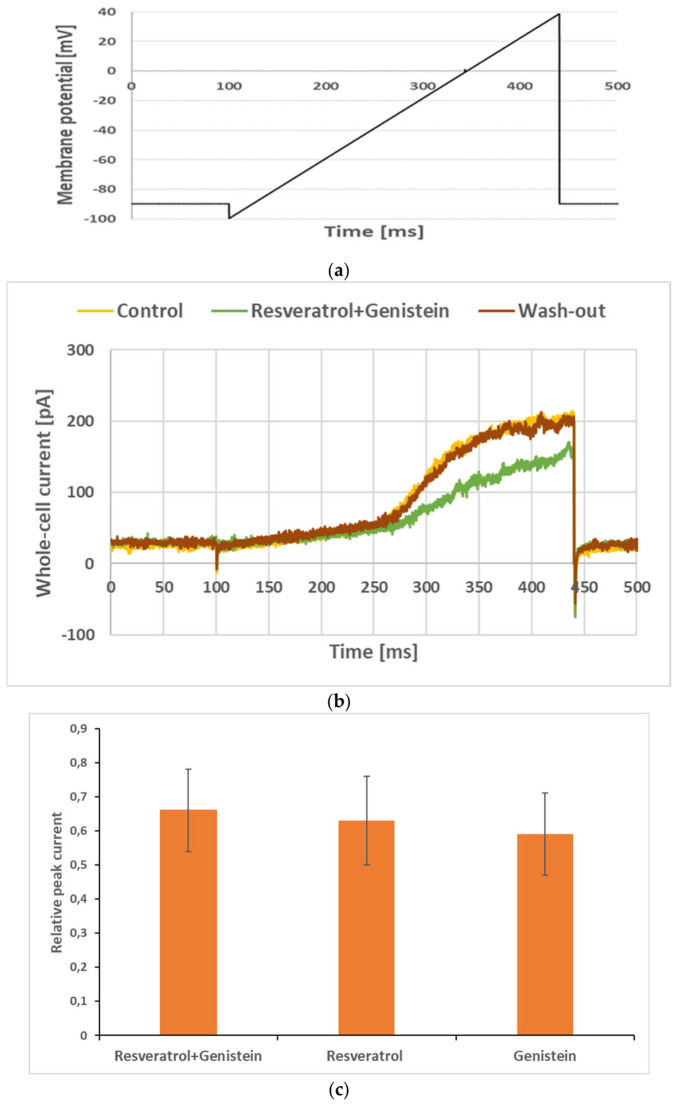
Whole-cell currents recorded in Jurkat T cells (**b**) by applying the “voltage ramp” (**a**) under control conditions upon co-application of 30 µM genistein with the same concentration of resveratrol during wash-out of the mixture (**b**). Relative peak currents upon co-application of genistein and resveratrol at a concentration of 30 µM and genistein and resveratrol applied at the same concentration alone (**c**). Error bars are shown.

Figure 6b shows whole-cell currents recorded under control conditions, upon co-application of genistein and resveratrol at 30 µM and after wash-out of the mixture.

The inhibitory effects upon the application of a mixture of two examined compounds could be additive, synergistic or non-additive [15]. In the case of an additive inhibitory effect (1 + 1 = 2), the relative peak current upon the application of a mixture is not significantly different (*p* > 0.05) from the product of multiple relative peak currents upon the application of each compound alone. In the case of a synergistic inhibitory effect (1 + 1 > 2), the relative peak current upon the application of a mixture is significantly (*p* < 0.05) lower than the product of multiple relative peak currents upon the application of each compound alone [17]. In the case of a non-additive inhibitory effect (1 + 1 < 2), the relative peak current upon the application of a mixture is significantly (*p* < 0.05) higher than the product of multiple relative peak currents upon the application of each compound alone [17].

The relative peak currents when genistein and resveratrol were applied alone at a 30 µM concentration were equal to 0.59 and 0.63, respectively (see above). The theoretical value of the relative peak current upon the co-application of genistein and resveratrol at this concentration is equal to 0.59 × 0.63 = 0.37. However, the whole-cell current recorded upon co-application of genistein and resveratrol was not significantly lower than the currents recorded upon the application of each compound alone (compare Figure 2 and Figure 6). In accordance with this observation, the calculated relative peak current upon co-application of genistein and resveratrol was equal to 0.66 ± 0.11 (n = 21). This value was not significantly different (*p* > 0.05) from the values obtained upon the application of each compound alone (Figure 6c) and was significantly (*p* < 0.05) higher than the theoretical value when assuming an additive inhibitory effect (Table 1). Thus, the inhibitory effects of genistein and resveratrol were not additive (Table 1).

Because the results of our previous studies showed that a co-application of some flavonoids and chalcones led to additive or synergistic inhibitory effects on the channels [17], the next goal of our study was to investigate whether a co-application of genistein and resveratrol with simvastatin and mevastatin led to an additive or synergistic inhibitory effect on the channels.

**Figure 7:**
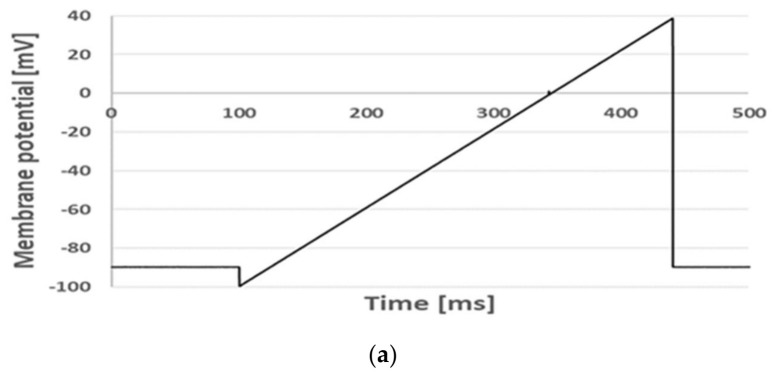

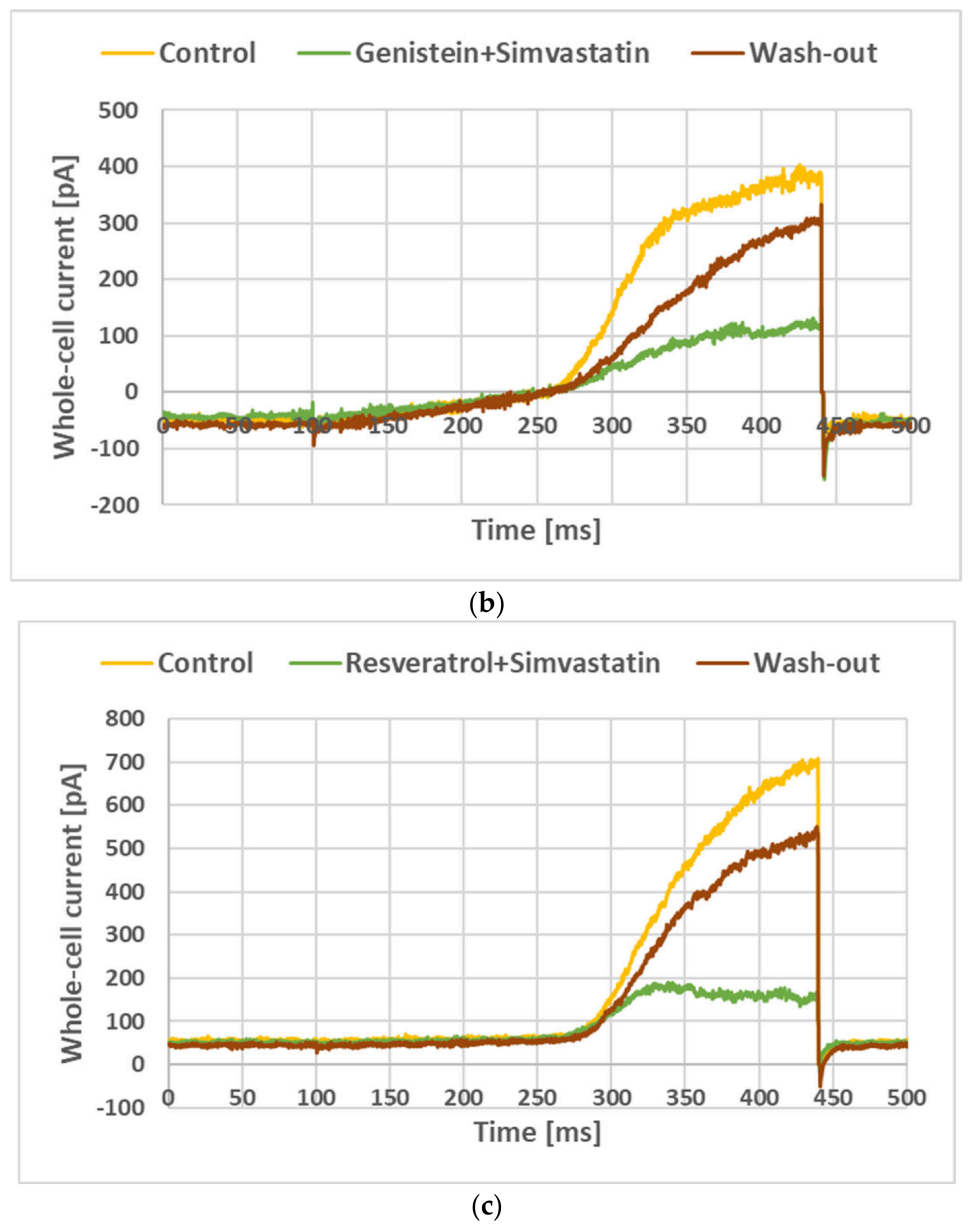
Whole-cell currents recorded in Jurkat T cells (**b**,**c**) applying the “voltage ramp” (**a**) under control conditions, upon co-application of 30 µM genistein with 6 µM of simvastatin; (**b**) 30 µM of resveratrol with 6 µM of simvastatin; (**c**) and after wash-out of the mixture.

Figure 7b shows whole-cell currents under control conditions upon co-application of genistein at a 30 µM concentration and simvastatin at a 6 µM concentration and after the mixture was washed out. Figure 7c depicts the currents recorded under control conditions, upon the co-application of resveratrol at a 30 µM concentration and simvastatin at a 6 µM concentration, and after wash-out of the mixture.

Apparently, the whole-cell currents recorded upon co-application of genistein and resveratrol with simvastatin were significantly lower than the currents recorded upon the application of either genistein or resveratrol alone (compare Figure 2 and Figure 7). Moreover, the currents incompletely recovered after the mixture was washed out. This may indicate that the inhibitory effect of the mixture on Kv1.3 channels is partially irreversible.

**Figure 8:**
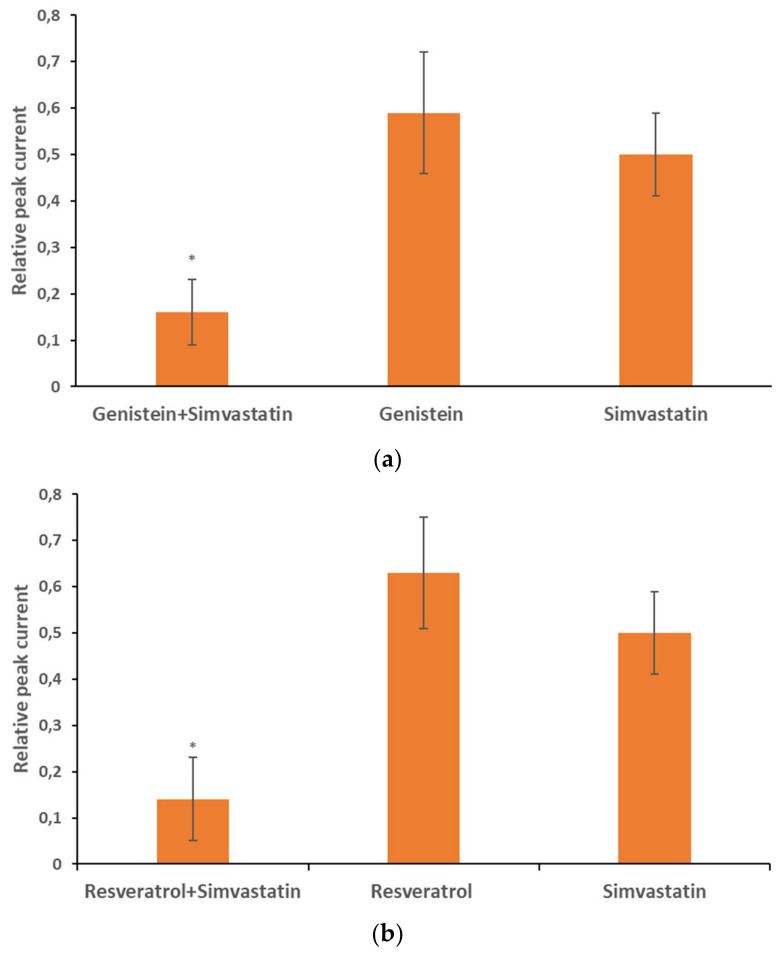
Relative peak current upon co-application of genistein at 30 µM concentration with simvastatin at 6 µM concentration; genistein at the same concentration applied alone; and simvastatin at the same concentration applied alone (**a**). Relative peak current upon co-application of resveratrol at 30 µM concentration with simvastatin at 6 µM concentration; resveratrol at the same concentration applied alone; and simvastatin at the same concentration applied alone (**b**). Error bars are shown. *—statistical significance.

Figure 8 shows relative peak currents calculated upon the co-application of 30 µM of genistein (a) and resveratrol (b) with 6 µM of simvastatin. For comparison, relative peak currents upon the application of each compound alone are also presented.

Upon co-application of genistein with simvastatin, the relative peak current was equal to 0.16 ± 0.07 (n = 32) compared to the control value. This value was significantly (*p* < 0.05) lower than the values upon application of genistein (this study) and simvastatin alone (0.50 ± 0.10, n = 16) [19]. The relative peak current during co-application was significantly (*p* < 0.05) lower than the product multiple currents recorded upon the application of each compound alone (Table 1). Thus, the inhibitory effect of genistein and simvastatin was synergistic (Table 1).

The relative peak current upon co-application of resveratrol and simvastatin was equal to 0.14 ± 0.09 (n = 17) of the control value. This value was significantly (*p* < 0.05) lower than the values upon application of resveratrol alone (this study) and simvastatin alone [19]. The relative peak current upon co-application was significantly (*p* < 0.05) lower than the product of multiplying the currents recorded upon the application of each compound alone (Table 1). Similarly, compared to the co-application of genistein and simvastatin together, the inhibitory effect of resveratrol and simvastatin was synergistic.

**Figure 9:**
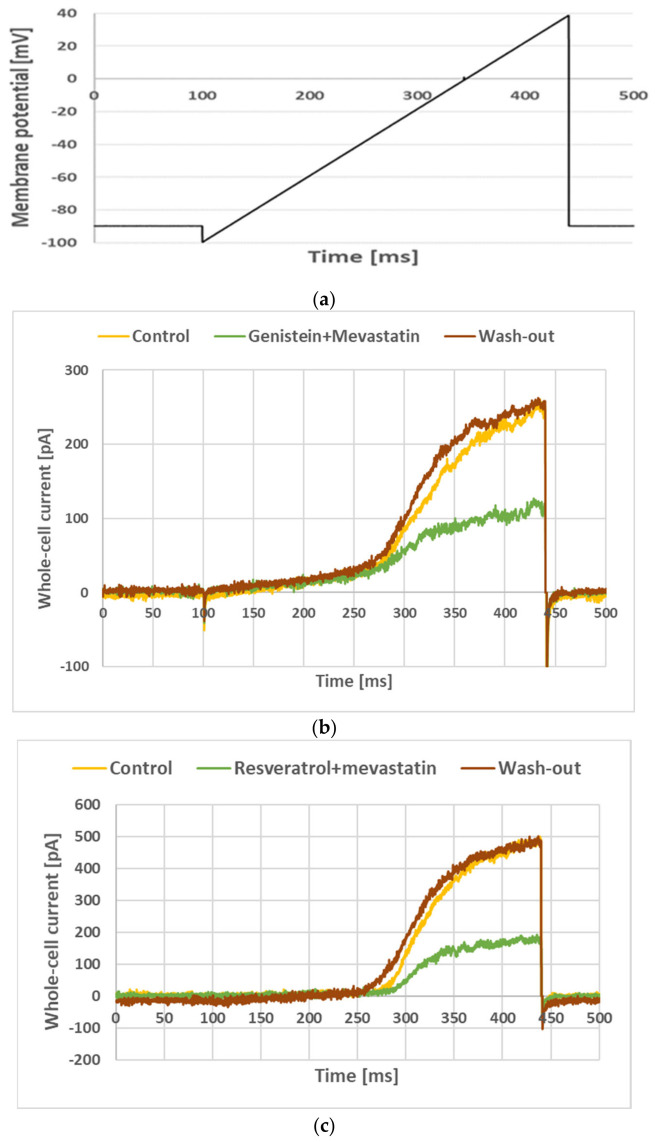
Whole-cell currents recorded in Jurkat T cells (**b**,**c**) after applying the “voltage ramp” (**a**) under control conditions upon co-application of 30 µM genistein with 6 µM of mevastatin, (**b**) 30 µM of resveratrol with 6 µM of mevastatin, and (**c**) and after wash-out of the mixture.

Figure 9b shows whole-cell currents under control conditions, upon the co-application of genistein at 30 µM concentration and mevastatin at 6 µM concentration and after wash-out of the mixture. Figure 9c depicts the current recorded under control conditions, upon co-application of resveratrol at a 30 µM concentration and mevastatin at a concentration of 6 µM and after wash-out of the mixture.

Apparently, the currents recorded upon a co-application of both compounds with mevastatin were significantly lower than the currents recorded upon an application of both genistein and resveratrol alone (compare Figure 2 and Figure 9). The currents recovered completely upon a wash-out of the mixture. This indicates that the inhibitory effect of the mixture on Kv1.3 channels was reversible.

**Figure 10:**
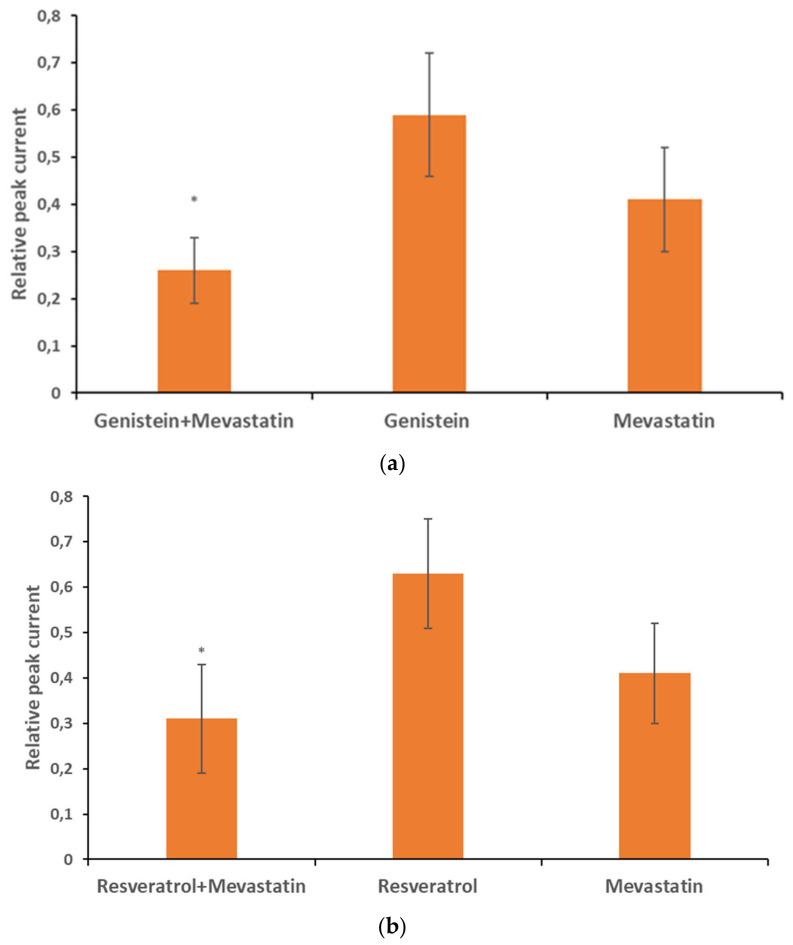
Relative peak current upon co-application of genistein at 30 µM concentration with mevastatin at 6 µM concentration; genistein at the same concentration applied alone; mevastatin at the same concentration applied alone (**a**). Relative peak current upon co-application of resveratrol at 30 µM concentration with mevastatin at 6 µM concentration; resveratrol at the same concentration applied alone; and mevastatin at the same concentration applied alone (**b**). Error bars are shown. *—statistical significance.

Figure 10 shows relative peak currents calculated upon co-application of 30 µM genistein (a) and resveratrol (b) with mevastatin applied at a concentration of 6 µM. For comparison, relative peak currents upon application of each compound alone were also presented. Upon application of the mixture, the relative peak current was equal to 0.26 ± 0.07 (n = 37) of the control value. This value was significantly (*p* < 0.05) lower than the values upon application of genistein alone (this study) and mevastatin alone (0.41 ± 0.10, n = 21) [19]. The relative peak current upon co-application was not significantly (*p* > 0.05) higher than the product of multiplication of the currents recorded upon the application of each compound alone (Table 1). This indicates that the inhibitory effects of genistein and mevastatin were additive (Table 1).

Upon co-application of resveratrol and mevastatin, the current was equal to 0.31 ± 0.12 (n = 31) of the control value. This value was significantly (*p* < 0.05) lower than the values upon application of resveratrol alone (this study) and mevastatin alone [19]. The relative peak current upon co-application was not significantly (*p* > 0.05) higher than the product of multiplication of the currents recorded upon the application of each compound alone (Table 1). This indicates that the inhibitory effects of resveratrol and mevastatin were additive (Table 1).

## 4. Discussion

The results presented in this paper may indicate that both genistein—a known isoflavone—and resveratrol—a polyphenolic compound—both inhibit Kv1.3 channels in Jurkat T cells dose-dependently but not completely, even at concentrations of 90 µM, where relative peak currents drop to half of the control value. An application of both compounds additionally causes a significant slowing of the current activation rate without significantly changing the inactivation rate. The inhibitory effects were not additive upon co-application of genistein and resveratrol. On the other hand, the inhibitory effects were additive when genistein and resveratrol were co-applied with mevastatin and synergistic when co-applied with simvastatin.

Our previous studies have shown that both genistein and resveratrol dose-dependently inhibit the activity of Kv1.3 channels in normal T lymphocytes [12]. The half-blocking concentration was about 40 µM for both compounds [12]. The channel activity was inhibited to about 0.23 of the control value in cases where genistein was applied at a concentration of 80 µM and about 0.25 upon application of resveratrol at a concentration of 100 µM [12]. These values were considerably lower than those calculated in this study for Kv1.3 channels in cancer cells.

This may indicate that channel inhibition in normal cells is stronger than observed in this study for cancer cells.

Similarly, to the observation in the case of Kv1.3 channels in cancer Jurkat T cells, the inhibition of channels in normal cells was accompanied by a significant slowing of the activation rate [12]. This may indicate that the putative mechanism of the channel inhibition remains the same in normal and cancer cells. It could be proposed that an application of genistein and resveratrol stabilized the channel proteins in the closed state, leading to a delay in their opening. Once the channel is open, the inhibitor’s molecules probably dissociate from the channel’s protein and rebind to the channel after its closure. Nevertheless, the extent of the channel inhibition is different. This may be due to the fact that the channels are expressed in different model systems. Different sensitivities of Kv1.3 channels expressed in different model systems for inhibition by the same compounds have widely been observed ([28], Gąsiorowska—unpublished observations).

Two major pathways can be used by inhibitors of Kv1.3 channels to interact with the channels’ proteins. The first, “extracellular” pathway is mainly used by membrane-impermeant peptide inhibitors. These compounds can bind to the extracellular vestibule of the channel and block the channel’s pore like a “cork in the bottle” [28]. The second, “intracellular” pathway can be used by small-molecule lipophilic compounds that share the ability to penetrate the cell membrane. These compounds may diffuse through the cell membrane and bind to their binding sites on the intracellular mouth of the channel [28]. It has been shown that the “intracellular” pathway is used by lovastatin and probably also by mevastatin and simvastatin [19,29]. It may be possible that the same pathway can also be used by genistein and resveratrol. In order to verify this hypothesis, more research studies need to be done.

According to our previous observations upon the co-application of flavonoids with statins, such as simvastatin and simvastatin, additive or synergistic inhibitory effects occurred [17,19]. More effective was the co-application with mevastatin [17,19]. In contrast to these observations, the results of this study show that the channel inhibition was more pronounced upon co-application with simvastatin. It was shown that the relative peak currents upon co-application with simvastatin were significantly lower than theoretical values calculated assuming that the inhibitory effects were additive. This indicates that these effects were synergistic. On the other hand, additive inhibitory effects were observed upon the co-application of genistein and resveratrol with mevastatin.

Additivity or synergism is important for the putative application of genistein and resveratrol to support cancer therapy. The co-applications improve on the modest inhibitory effect exerted by genistein and resveratrol when applied alone. This may enable a reduction in the putative required therapeutic dose and minimize the risk of unwanted side effects [15,17].

Interestingly, the inhibitory effect upon co-applications with simvastatin was partially irreversible. Our previous study showed that an application of simvastatin caused a partially irreversible inhibition of Kv1.3 channels in Jurkat T cells [19]. Irreversibility may be due to irreversible perturbations in structure of membrane lipid bilayers. It was shown that an application of simvastatin caused an irreversible decrease in the membrane capacitance [30]. This decrease was probably a consequence of an irreversible increase in membrane thickness, probably due to accumulation of simvastatin in the plasma membrane [30]. Accumulated drug molecules may directly or indirectly interact with channel proteins, irreversibly inhibiting the channel [30]. Results of this study have shown that partial irreversibility of the inhibitory effect occurred upon the co-application of simvastatin at a 6 µM concentration with genistein or resveratrol. On the other hand, our previous study showed that an application of simvastatin alone at the concentrations of 6 µM and 7.5 µM caused a significant, but reversible inhibitory effect on the channels [19]. This partial irreversibility observed in this study upon co-application could be a consequence of synergistic interactions of simvastatin combined with genistein or resveratrol with membrane lipid bilayers, leading to irreversible perturbations in its structure.

Channel inhibition may be related to anti-cancer activities of genistein and resveratrol [1,2]. It is known that lipophilic inhibitors of Kv1.3 channels may simultaneously inhibit uncontrolled proliferation of Kv1.3 channel-expressing cancer cells and induce the mitochondrial pathway of apoptosis for these cells [5,6,7,8,9,10,11,12]. The inhibition of proliferation may be related to an inhibition of plasma membrane Kv1.3 channels, whereas apoptosis induction may be a result of the inhibition of Kv1.3 channels in the inner mitochondrial membrane (mito Kv1.3 channels) [5,6,7,8,9,10,11,12]. It is known that genistein inhibits proliferation in Kv1.3 channel-expressing breast cancer cell lines of MCF-7 and MDA-MB- 231 [31], the colon cancer cell line HT-29 [32] and induces apoptosis in the colon cancer cell line HT-29 [32]. Genistein is considered a putatively potent anti-breast cancer agent [33,34]. Resveratrol inhibits proliferation and induces apoptosis of the Kv1.3 channel-expressing colon cancer cell line Caco-2 [35]. It also induces apoptosis of Kv1.3 channel-expressing U937 and MOLT-4 leukemia cells, MCF-7 breast cancer cells and A549 lung cancer cells [36].

Importantly, some lipophilic inhibitors of Kv1.3 channels, in particular “mitochondriotropic” compounds (drugs that preferentially bind to mitoKv1.3 channels), share the ability to selectively induce apoptosis of Kv1.3 channel-expressing cancer cells while sparing normal cells, even if normal cells express a large number of Kv1.3 channels [7,8,9,10,11,12,15,16,17,18,19,20]. This is due both to an up-regulation of Kv1.3 channels in some cancer disorders and increased basal production of reactive oxygen species (ROS) by cancer cell mitochondria. The combination of both factors activates the mitochondrial pathway of cancer cell apoptosis. Normal cells do not undergo apoptosis in such a way because of a low basal level of ROS [7,8,9,10,11,12,15,16,17,18,19,20].

It may be possible that these anti-proliferative and pro-apoptotic effects of both compounds may be, at least partially, due to the inhibition of mito Kv1.3 channels in Kv1.3 channel-expressing cancer cells, leading to the selective apoptosis of Kv1.3 channel-expressing cancer cells. More research is necessary to verify this hypothesis.

Altogether, our results indicate that both genistein and resveratrol may be applied to support chemotherapy of cancer disorders, with an over-expression of Kv1.3 channels [6,7,8,9,10,11,12,15,16,17,18,19,20], especially when co-applied with simvastatin and mevastatin. For this purpose, more studies are necessary to provide evidence that an application of genistein and resveratrol with and without statins leads to the selective elimination of cancer cells without killing normal ones, which occurs upon the application of “mitochondriotropic” inhibitors of mito Kv1.3 channels [7,8,9,10,11,12,15,16,17,18,19,20].

## 5. Conclusions

The results of this study show that both genistein and resveratrol inhibit Kv1.3 channels in Jurkat T cancer cells. The mechanism of inhibition is probably the same in normal cells and cancer cells. Inhibition of the channels in cancer cells by genistein and resveratrol is weaker than in normal cells. On the other hand, channel inhibition in cancer cells is significantly ameliorated upon co-application of these compounds with simvastatin and mevastatin. Channel inhibition may putatively be involved in anti-cancer activity of these compounds; however, more research studies are necessary to resolve this problem.

## Figures and Tables

**Table 1:** Comparisons of theoretical and experimental relative peak currents upon co-application of genistein with resveratrol and co-application of each compound with simvastatin and mevastatin. *—Experimental values significantly higher; **—experimental values significantly lower than theoretical ones; no asterisk next to experimental values—no significant difference from theoretical values.

Combination of the Compounds/ Concentration	Relative Peak Currents—Theoretical Values	Relative Peak Currents—Experimental Values	Channel Inhibition upon a Co-Application
Genistein [30 μM] + Resveratrol [30 μM]	0.37	0.66 *	Not additive
Genistein [30 μM] + Simvastatin [6 μM]	0.295	0.16 **	Synergistic
Resveratrol [30 μM] + Simvastatin [6 μM]	0.315	0.14 **	Synergistic
Genistein [30 μM] + Mevastatin [6 μM]	0.24	0.26	Additive
Resveratrol [30 μM] + Mevastatin [6 μM]	0.26	0.31	Additive

## Data Availability

The data that support the findings of this study are available from the first author, Andrzej Teisseyre, upon reasonable request.

## Supplementary Materials

The following supporting information can be downloaded at https://www.mdpi.com/article/10.3390/membranes16050159/s1. Supplementary Table S1. Relative peak current upon an application of genistein at various concentrations; Supplementary Table S2. Relative peak current upon an application of resveratrol at various concentrations; Supplementary Table S3. Mean time-to-peak values prior to application (Control) and upon an application of genistein at given concentrations; Supplementary Table S4. Mean time-to-peak values prior to application (Control) and upon an application of resveratrol at given concentrations; Supplementary Table S5. Inactivation time constant (τ) values prior to application (Control) and upon an application of genistein and resveratrol at given concentrations; and Supplementary Table S6. Relative peak current upon an co-application of combinations of examined compounds.

## Abbreviations

The following abbreviations are used in this manuscript:

Kv1.3	voltage-gated potassium channel studied
I	Kv1.3 current upon application of an examined compound at +40 mV
I_contr_	Kv1.3 current recorded on the same cell at +40 mV under control conditions
T_peak_	time to peak
RKS	relative kinetics slowing
T_peakcompound_	mean time-to-peak value upon application of a compound
T_peakcontrol_	mean time-to-peak value under control conditions
τ	inactivation time constant

## References

[1] Naeem H., Momal U., Imran M., Shahbaz M., Hussain M., Alsagaby S.A., Al Abdulmonem W., Umar M., Mujtaba A., El-Ghorab A.H. (2023). Anticancer perspectives of genistein: A comprehensive review. Int. J. Food Prop..

[2] Richter-Laskowska M., Trybek P., Delfino D.V., Wawrzkiewicz-Jałowiecka A. (2023). Flavonoids as modulators of potassium channels. Int. J. Mol. Sci..

[3] Kursvietiene L., Kopustinskiene D.M., Staneviciene I., Mongirdiene A., Kubová K., Masteikova R., Bernatoniene J. (2023). Anti-cancer properties of Resveratrol: A focus on its impact on mitochondrial functions. Antioxidants.

[4] Gutman G.A., Chandy K.G., Grissmer S., Lazdunski M., Mckinnon D., Pardo L.A., Robertson G.A., Rudy B., Sanguinetti M.C., Stühmer W. (2005). International Union of Pharmacology. LIII. Nomenclature and Molecular Relationships of Voltage-gated Potassium channels. Pharmacol. Rev..

[5] Gulbins E., Sassi N., Grassme H., Zoratti M., Szabo I. (2010). Role of Kv1.3 mitochondrial potassium channel in apoptotic signalling in lymphocytes. Biochim. Biophys. Acta-Bioenerg..

[6] Perez-Verdaguer M., Capera J., Serrano-Novillo C., Estadella I., Sastre D., Felipe A. (2016). The voltage-gated potassium channel Kv1.3 is a promising multitherapeutic target against human pathologies. Expert Opin. Ther. Targets.

[7] Leanza L., Romio M., Becker K.A., Azzolini M., Trentin L., Manago A., Venturini E., Zaccagnino A., Mattarei A., Carraretto L. (2017). Direct pharmacological targeting of a mitochondrial ion channel selectively kills tumor cells In Vivo. Cancer Cell.

[8] Peruzzo R., Mattarei A., Romio M., Paradisi C., Zoratti M., Szabò I., Leanza L. (2017). Regulation of proliferation by a mitochondrial potassium channel in pancreatic ductal adenocarcinoma cells. Front. Oncol..

[9] Serrano-Albarras A., Estadella I., Cirera-Rocosa S., Navarro-Perez M., Felipe A. (2017). Kv1.3: A multifunctional channel with many pathological implications. Expert Opin. Ther. Targets.

[10] Prosdocimi E., Checchetto V., Leanza L. (2019). Targeting the mitochondrial potassium channel Kv1.3 to kill cancer cells: Drugs, strategies and new perspectives. SLAS Discov..

[11] Checchetto V., Prosdocimi E., Leanza L. (2019). Mitochondrial Kv1.3: A new target in cancer biology?. Cell. Physiol. Biochem..

[12] Teisseyre A., Palko-Labuz A., Środa-Pomianek K., Michalak K. (2019). Voltage-gated potassium channel Kv1.3 as a target in therapy of cancer. Front. Oncol..

[13] Fan C., Yang X., Wang W., Wang J., Li W., Guo W., Huang S., Wang Z., Liu K. (2020). Role of Kv1.3 channels in platelet functions and thrombus formation. Arterioscler. Thromb. Vasc. Biol..

[14] Bobi J., Garabito M., Solanes N., Cidad P., Ramos-Perez V., Ponce A., Rigol M., Freixa X., Pérez-Martínez C., de Prado A.P. (2020). Kv1.3 blockade inhibits proliferation of vascular smooth muscle cells in vitro and intimal hyperplasia in vivo. Transl. Res..

[15] Kadow S., Schumacher F., Kramer M., Hessler G., Scholtysik R., Oubari S., Johansson P., Hüttmann A., Reinhardt H.C., Kleuser B. (2022). Mitochondrial Kv1.3 channels as Target for Treatment of Multiple Myeloma. Cancers.

[16] Severin F., Urbani A., Valanita T., Bachmann M., Azzolini M., Martini V., Pizzi M., Tos A.P.D., Frezzato F., Mattarei A. (2022). Pharmacological modulation of Kv1.3 potassium channel selectively triggers pathological B lymphocyte apoptosis in vivo in a genetic CLL model. J. Exp. Clin. Cancer Res..

[17] Teisseyre A., Chmielarz M., Uryga A., Środa-Pomianek K., Palko-Labuz A. (2022). Co-application of statin and flavonoids as an effective strategy to reduce the activity of voltage-gated potassium channels Kv1.3 and induce apoptosis in human leukemic T cell line Jurkat. Molecules.

[18] Patel S., Bachmann M., Kadow S., Wilson G., Abdel-Salam M., Xu K., Keitsch S., Soddemann M., Wilker B., Becker K.A. (2023). Simultaneous targeting of mitochondrial Kv1.3 and lysosomal acid sphingomyelinase amplifies killing of pancreatic ductal adenocarcinoma cells in vitro and in vivo. J. Mol. Med..

[19] Teisseyre A., Środa-Pomianek K., Palko-Łabuz A. (2024). The influence of naturally occurring flavonoids, chalcones and statins on the activity of voltage-gated potassium channels Kv1.3 and viability of Kv1.3 channel-expressing cancer cells. J. Mol. Struct..

[20] Cheng S., Jiang D., Lan X., Liu K., Fan C. (2024). Voltage-gated potassium channel 1.3: A promising molecular target in multiple disease therapy. Biomed. Pharmacother..

[21] Chen Y., Zhi Y., Zhong H., Ma L., Gu X., Cai Y., Huang J., Yi X., Wu X., Yung K.K.L. (2025). Inhibition of Kv1.3 channel restrains macrophage M2 polarization and ameliorates renal fibrosis via regulating STAT6 phosphorylation. Pharmacol. Res..

[22] Attali B., Romey G., Honore E., Schmid-Alliana A., Mattei M., Lesage F., Ricard P., Barhanin J., Lazdunski M. (1992). Cloning, functional expression, and regulation of two K^+^ channels in human T lymphocytes. J. Biol. Chem..

[23] Valencia-Cruz G., Shabala L., Delgado-Enciso I., Shabala S., Bonales-Alatorre E., Pottosin I., Dobrovinskaya O. (2009). K_bg_ and Kv1.3 channels mediate potassium efflux in the early phase of apoptosis in Jurkat T lymphocytes. Am. J. Physiol.-Cell Physiol..

[24] Grissmer S., Nguyen A., Cahalan M. (1993). Calcium-activated potassium channels in resting and activated human T lymphocytes. J. Gen. Physiol..

[25] Grissmer S., Lewis R., Cahalan M. (1992). Ca^2+^-activated K^+^ Channels in Human Leukemic T Cells. J. Gen. Physiol..

[26] Hamill O., Marty A., Neher E., Sakmann B., Sigworth F. (1981). Improved patch-clamp techniques for high-resolution current recording from cells and cell-free membrane patches. Pflügers Arch..

[27] Teisseyre A., Mozrzymas J. (2002). Inhibition of the Activity of T lymphocyte Kv1.3 Channels by Extracellular Zinc. Biochem. Pharmacol..

[28] Panyi G., Possani L., Rodriguez de la Vega R., Gaspar R., Varga Z. (2006). K^+^ channel blockers: Novel tools to inhibit T cell activation leading to specific immunosuppression. Curr. Pharm. Des..

[29] Zhao N., Dong Q., Qian C., Li S., Wu Q., Ding D., Li J., Wang B.-B., Guo K.-F., Xie J.-J. (2015). Lovastatin blocks Kv1.3 channel in human T cells: A new mechanism to explain its immunomodulatory properties. Sci. Rep..

[30] Kazama I., Baba A., Maruyama Y. (2014). HMG-CoA reductase inhibitors: Pravastatin, lovastatin and simvastatin suppress delayed rectifier K^+^-channel currents in murine thymocytes. Pharmacol. Rep..

[31] Shon Y., Park S., Nam K. (2006). Effective chemopreventive activity of genistein against human breast cancer cells. BMB Rep..

[32] Yu Z., Li W., Liu F. (2004). Inhibition of proliferation and induction of apoptosis by genistein in colon cancer HT-29 cells. Cancer Lett..

[33] Bhat S.S., Prasad S.K., Shivamallu C., Prasad K.S., Syed A., Reddy P., Cull C.A., Amachawadi R.G. (2021). Genistein: A potent anti-breast cancer agent. Curr. Issues Mol. Biol..

[34] Pawlicka M.A., Zmorzyński S., Popek-Marciniec S., Filip A.A. (2022). The effects of genistein at different concentrations on MCF-7 breast cancer cells and BJ dermal fibroblasts. Int. J. Mol. Sci..

[35] Fouad M., Agha A., Al Merzabani M., Shouman S. (2013). Resveratrol inhibits proliferation, angiogenesis and induces apoptosis in colon cancer cells: Calorie restriction is the force to the cytotoxicity. Hum. Exp. Toxicol..

[36] Takashina M., Inoue S., Tomihara K., Tomita K., Hattori K., Zhao Q.-L., Suzuki T., Noguchi M., Ohashi W., Hattori Y. (2017). Different effect of resveratrol to induction of apoptosis depending on the type of human cancer cells. Int. J. Oncol..

