# Supplementary material for: Genistein and Resveratrol: Inhibitors of Kv1.3 Channels in Cancer Cells
Source: Membranes (Basel). 2026 Apr 30;16(5):159. doi: 10.3390/membranes16050159 (PMC13208166; doi:10.3390/membranes16050159)
Supplement: Supplementary file 1 [file membranes-16-00159-s001.zip › membranes-4245102-supplementary.pdf]

Supplementary Table 1. Relative peak current upon an application of genistein at various concentrations.

Concentration of Genistein [µM]	Relative peak current upon the drug application
3	0,72; 0,88; 0,86; 0,82; 0,80; 0,96; 0,82; 0,36; 0,86; 0,86; 0,61; 0,72; 0,42; 0,84; 0,98; 0,95; 0,88
6	0,90; 0,99; 0,94; 0,92; 0,80; 0,96; 0,66; 0,74; 1,00; 0,61; 0,83; 0,61; 0,90; 0,83; 0,85; 0,93; 0,88; 0,77
12	0,56; 0,51; 0,81; 0,81; 0,97; 0,75; 0,86; 0,88; 0,50; 0,83; 0,79; 0,84; 0,82; 0,54; 0,74; 0,76; 0,89; 0,79; 0,92; 0,49; 0,86
15	0,85; 0,71; 0,65; 0,72; 0,78; 0,81; 0,82; 0,70; 0,76; 0,78; 0,73; 0,44; 0,78; 0,90; 0,76; 0,72; 0,22; 0,30;
30	0,60; 0,68; 0,42; 0,63; 0,64; 0,75; 0,61; 0,64; 0,63; 0,61; 0,65; 0,64; 0,72
60	0,57; 0,65; 0,56; 0,67; 0,68; 0,40; 0,64; 0,61; 0,19; 0,61; 0,12; 0,37; 0,56; 0,54; 0,56; 0,26; 0,61; 0,52; 0,54; 0,54; 0,61
90	0,44; 0,57; 0,61; 0,67; 0,40; 0,56; 0,53; 0,47; 0,55; 0,34; 0,49; 0,44; 0,45; 0,35;, 0,62; 0,47; 0,41; 0,40; 0,52; 0,65

Supplementary Table 2. Relative peak current upon an application of resveratrol at various concentrations.

Concentration of Resveratrol [µM]	Relative peak current upon the drug application
3	0,97; 0,93; 0,93; 1,01; 0,92; 0,95; 0,96; 0,84; 0,89; 0,82; 0,72; 0,83; 0,85; 0,94; 0,84; 0,93; 0,90; 0,79; 0,97
4,5	0,96; 0,94; 0,95; 0,95; 0,95; 0,86; 0,52; 0,95; 0,99; 0,72; 0,55; 1,05; 0,97; 1,02; 0,81; 0,91; 0,89; 0,75; 0,79
7,5	0,77; 0,85; 0,98; 0,73; 0,74; 0,82; 0,84; 0,85; 0,72; 0,89; 0,80; 0,82; 0,78; 0,80; 0,86; 0,91; 0,98; 0,88; 0,70; 0,92; 0,87
15	0,77; 0,86; 0,84; 0,75; 0,73; 0,63; 0,68; 0,76; 0,72; 0,73; 0,71; 0,67; 0,53; 0,64; 0,90; 0,79; 0,74; 0,82; 0,71; 0,84; 0,87; 0,66; 0,74; 0,65
30	0,76; 0,61; 0,63; 0,68; 0,69; 0,61; 0,72; 0,77; 0,59; 0,49; 0,36
60	0,56; 0,52; 0,40; 0,51; 0,67; 0,56; 0,39; 0,65; 0,62; 0,48; 0,31
90	0,43; 0,55; 0,48; 0,44; 0,30; 0,39; 0,58; 0,47; 0,48; 0,49; 0,44; 0,48; 0,48; 0,39; 0,44; 0,46; 0,49; 0,61; 0,39; 0,59

Supplementary Table 3. Mean time-to-peak values prior to application (Control) and upon an application of genistein at given concentrations.

Experimental conditions	$T_{peakcontrol}$ [ms]	Experimental conditions	$T_{peakcompound}$ [ms]
Control	9.7±1.17 (n=5)	Genistein 3 μM	14.72±4.09 (n=5)
Control	9.58±2.51 (n=5)	Genistein 6 μM	14.56±3.08 (n=5)
Control	12.23±2.46 (n=8)	Genistein 12 μM	25.26±5.47 (n=8)
Control	8.19±1.54 (n=7)	Genistein 15 μM	20.10±2.84 (n=7)
Control	8.97±1.50 (n=8)	Genistein 30 μM	20.59±5.78 (n=8)
Control	8.81±2.49 (n=20)	Genistein 60 μM	25.71±7.57 (n=20)
Control	7.95±1.19 (n=20)	Genistein 90 μM	30.17±4.43 (n=20)

Supplementary Table 4. Mean time-to-peak values prior to application (Control) and upon an application of resveratrol at given concentrations.

Experimental conditions	$T_{peakcontrol}$ [ms]	Experimental conditions	$T_{peakcompound}$ [ms]
Control	8.29±1.42 (n=29)	Resveratrol 3 µM	8.21±1.32 (n=34)
Control	10.20±0.83 (n=9)	Resveratrol 4.5 µM	10.20±1.04 (n=8)
Control	8.58±1.75 (n=17)	Resveratrol 7.5 µM	9.23±2.15 (n=16)
Control	12.58±1.25 (n=4)	Resveratrol 15 µM	14.77±1.86 (n=3)
Control	7.36±2.09 (n=26)	Resveratrol 30 µM	9.10±2.10 (n=26)
Control	6.99±2.07 (n=12)	Resveratrol 60 µM	14.10±2.77 (n=12)
Control	6.99±1.18 (n=16)	Resveratrol 90 µM	14.79±3.02 (n=20)

Supplementary Table 5. Inactivation time constant (τ) values prior to application (Control) and upon an application of genistein and resveratrol at given concentrations.

Experimental conditions	Inactivation time constants (τ) [ms]	Experimental conditions	Inactivation time constants (τ) [ms]
Control	253.9; 183.1; 165.0; 193.3; 159.4; 213.4; 200.2; 180.7; 151.7; 263.0; 176.6; 197.7; 279.6; 259.2; 225.1; 226.4;	Genistein 30 μM	201.4; 160.5; 163.9; 216.4; 183.6; 169.2; 208.5; 184.6; 204.6; 168.0; 238.0; 177.7; 242.7; 197.6; 221.3; 218.1;
Control	175.1; 174.7; 195.1; 136.5; 159.4; 206.6; 179.0; 225.2; 196.1; 157.1; 176.1; 138.2; 161.8; 143.6; 172.7; 209.2;	Resveratrol 90μM	183.6; 150.1; 318.4; 197.1; 191.6; 201.5; 212.3; 199.4; 113.3; 169.9; 210.6; 203.3; 175.5; 126.2;

Supplementary Table 6. Relative peak current upon an co-application of combinations of examined compounds.

Combination of the compounds/ Concentration	Relative peak current upon the combination application
Genistein 30 μM + Resveratrol 30 μM	0,57; 0,69; 0,62; 0,66; 0,67; 0,46; 0,76; 0,82; 0,81; 0,64; 0,75; 0,70; 0,66; 0,51; 0,79; 0,65; 0,78; 0,55; 0,66; 0,44; 0,67;
Genistein 30 μM + Simvastatin 6 μM	0,10; 0,11; 0,14; 0,07; 0,08; 0,08; 0,16; 0,18; 0,33; 0,07; 0,08; 0,12; 0,16; 0,14; 0,15; 0,20; 0,25; 0,22; 0,22; 0,22; 0,26; 0,17; 0,10; 0,10; 0,07; 0,19; 0,11; 0,14; 0.19; 0,23; 0,16; 0,31
Resveratrol 30 μM + Simvastatin 6 μM	0,20; 0,07; 0,06; 0,09; 0,04; 0,20; 0,10; 0,43; 0,12; 0,12; 0,17; 0,17; 0,14; 0,10; 0,11; 0,13; 0,10
Genistein 30 μM + Mevastatin 6 μM	0,23; 0,26; 0,34; 0,29; 0,17; 0,10; 0,27; 0,24; 0,40; 0,31; 0,29; 0,27; 0,33; 0,30; 0,36; 0,24; 0,30; 0,20; 0,22; 0,19; 0,16; 0,36; 0,17; 0,25; 0,19; 0,26; 0,21; 0,30; 0,13; 0,33; 0,21; 0,28; 0,18; 0,26; 0,28; 0,38; 0,24;
Resveratrol 30 μM + Mevastatin 6 μM	0,47; 0,30; 0,23; 0,09; 0,38; 0,42; 0,31; 0,34; 0,34; 0,16; 0,19; 0,18; 0,33; 0,44; 0,51; 0,18; 0,30; 0,51; 0,48; 0,15; 0,45; 0,39; 0,44; 0,30; 0,31; 0,35; 0,24; 0,22; 0,39; 0,20, 0,15
